# Advanced hemodynamics for prognostication in heart failure: the pursuit of the patient-specific tipping point

**DOI:** 10.3389/fcvm.2024.1365696

**Published:** 2024-03-04

**Authors:** Jonathan Grinstein

**Affiliations:** Department of Medicine, Section of Cardiology, University of Chicago, Chicago, IL, United States

**Keywords:** hemodynamic, prognosticating evaluation, energetics, tipping point dynamics, heart failure

## Abstract

**Background:**

Objective tools to define the optimal time for referral for advanced therapies and to help guide escalation and de-escalation of support can improve management decisions and outcomes for patients with advanced heart failure. The current parameters have variable prognostic potential depending on the patient population being studied and often have arbitrary thresholds.

**Methods:**

Here, a mathematical and physiological framework to define the patient-specific tipping point of myocardial energetics is defined. A novel hemodynamic parameter known as the myocardial performance score (MPS), a marker of power and efficiency, is introduced that allows for the objective assessment of the physiological tipping point. The performance of the MPS and other advanced hemodynamic parameters including aortic pulsatility index (API) and cardiac power output (CPO) in predicting myocardial energetics and the overall myocardial performance was evaluated using a validated computer simulation model of heart failure (Harvi) as well as a proof-of-concept clinical validation using a cohort of the Society for Cardiovascular Angiography and Interventions (SCAI) Stage C cardiogenic shock patients.

**Results:**

Approximately 1010 discrete heart failure scenarios were modeled. API strongly correlated with the left ventricular coupling ratio (*R*^2^ = 0.81) and the strength of association became even stronger under loaded conditions where pulmonary capillary wedge pressure (PCWP) was >20 mmHg (*R*^2^ = 0.94). Under loaded conditions, there is a strong logarithmic relationship between MPS and mechanical efficiency (*R*^2^ = 0.93) with a precipitous rise in potential energy (PE) and drop in mechanical efficiency with an MPS <0.5. An MPS <0.5 was able to predict a CPO <0.6 W and coupling ratio of <0.7 with sensitivity (Sn) of 87%, specificity (Sp) of 91%, positive predictive value of 81%, and negative predictive value of 94%. In a cohort of 224 patients with SCAI Stage C shock requiring milrinone initiation, a baseline MPS score of <0.5 was associated with a 35% event rate of the composite endpoint of death, left ventricular assist device, or transplant at 30 days compared with 3% for those with an MPS >1 (*p* < 0.001). Patients who were able to augment their MPS to >1 after milrinone infusion had a lower event rate than those with insufficient reserve (40% vs. 16%, *p* = 0.01).

**Conclusions:**

The MPS, which defines the patient-specific power-to-efficiency ratio and is inversely proportional to PE, represents an objective assessment of the myocardial energetic state of a patient and can be used to define the physiological tipping point for patients with advanced heart failure.

## Introduction

Timely referral for consideration of advanced therapies is of the upmost importance for patients with advanced heart failure and cardiogenic shock given the rapid and unpredictable progression of the disease. The hemodynamic assessment plays a central role in risk stratification in heart failure; however, a fundamental limitation of our current utilization of hemodynamic data is our reliance on thresholds for each parameter that are sometimes arbitrary [i.e., pulmonary capillary wedge pressure (PCWP) >15 or 18 mmHg, CI <2.2 or 2.0 or 1.8 L/min/m^2^] and often poorly defined across the continuity of heart failure. For certain parameters, discrete thresholds have been set; however, these thresholds are typically statistical in nature (not physiological thresholds) defined using receiver-operator-characteristic analysis from a very specific patient population that may or may not be representative of a given patient under a provider's care (1).

The routine use of continuous hemodynamic monitoring, for both risk assessment and management, has had a tumultuous history. Pulmonary artery catheters (PAC) have been utilized for real-time hemodynamic monitoring dating back to the 1970s; however, their use abruptly declined following the publication of the ESCAPE trial in the early 2000s, which failed to show a survival benefit with routine PAC use (2, 3). More recently, there has been a resurgence of PAC use following emerging data that a complete invasive hemodynamic assessment confers a survival benefit in the modern era (4–6).

Despite the resurgence of routine hemodynamic monitoring, the standard hemodynamic assessment, where intracardiac filling pressures and cardiac output are measured, has variable prognostic performance. While elevated filling pressures routinely confer a poor prognosis, a low cardiac output or cardiac index has had more inconstant prognostic certainty (7–10). To overcome the limitations of standard hemodynamic parameters, advanced hemodynamic parameters were derived to better reflect the interaction of loading conditions and cardiac performance. The left-sided advanced parameters of cardiac power output (CPO), aortic pulsatility index (API), and left ventricular stroke work index (LVSWI) have improved prognostic performance over standard hemodynamic parameters (8, 9, 11–13). Similarly, the right-sided advanced parameters of pulmonary artery pulsatility index (PAPI) and right ventricular stroke work index (RVSWI) have an important role in predicting and monitoring for post-operative right ventricular dysfunction (14–18).

Although the prognostic performance of the hemodynamic assessment has improved with the incorporation of advanced hemodynamic parameters, we continue to rely on statistical thresholds, which were derived at the population level, to help guide care. For us to maximize the predictive capabilities of the hemodynamic assessment, we need to move away from arbitrary thresholds and instead focus on the physiological tipping points. By doing so, patient-specific thresholds, rather than statistical, population-based thresholds can be defined, which would be more informative for the individual patient.

Here, the physiological underpinnings of the advanced hemodynamic parameters are first defined and the interplay of hemodynamics and energetics is introduced to define the true physiological tipping point for the cardiovascular system. The unique physiological properties of this tipping point are further explored, and a user-friendly hemodynamic monitoring parameter known as the myocardial performance score (MPS) is introduced to help define the hemometabolic state of a patient and their patient-specific tipping point.

## Methods

### Validation using simulated patients

To test the performance of the advanced hemodynamic variables in relationship to myocardial energetics and the physiological tipping point, a validated computer simulation model (Harvi Cardiovascular Simulation, PV Loops, LLC) was used. A total of 1,010 clinical scenarios of heart failure were generated by sequentially varying preload (*n* = 7), afterload (*n* = 7), contractility (*n* = 6), and heart rate (HR) (*n* = 3) as previously defined (13). Outputs including systemic blood pressure, intracardiac filling pressures, cardiac output, ventricular elastance (end-systolic elastance, Ees), aortic elastance (effective arterial elastance, Ea), and cardiac energetics [stroke work (SW) and potential energy (PE)] were recorded for each simulated heart failure scenario. Advanced hemodynamic parameters were derived for each scenario. Cardiac power output was calculated as [mean arterial pressure (MAP) × cardiac output]/451. API was calculated as [systolic blood pressure (SBP) − diastolic blood pressure (DBP)]/PCWP. The novel parameter, the MPS, was calculated as (API × CPO)/2. Regression models were generated to determine the relationship of the hemodynamic variables to cardiac energetics, and the coefficient of determination (*R*^2^) for each relationship was derived. Sensitivity (Sn), specificity (Sp), positive predictive values (PPV), and negative predictive values (NPV) were determined for variables of interest. To further define the relationship of these variables at the extremes of cardiac performance, the simulated scenarios were restricted to scenarios resulting in a PCWP >20 mmHg when denoted.

### Clinical validation

As a proof of concept, the performance of the MPS was studied using a cohort of 224 patients with American College of Cardiology and the American Heart Association (ACC/AHA) Stage D heart failure, the Society for Cardiovascular Angiography and Interventions (SCAI) Stage C shock. This study was approved by the University of Chicago Institutional Review Board. Details of this cohort have been previously defined (8). All patients included in this analysis had baseline hemodynamics that were consistent with a low output (CI <2.2 L/min/m^2^), congested state (PCWP >15 mmHg) warranting initiation of milrinone. All patients had hemodynamics assessed at baseline and again 10 min after a milrinone load of 50 μg/kg/min. Both static and dynamic changes in advanced hemodynamic parameters were analyzed. The composite clinical endpoint was death, left ventricular assist device (LVAD), or transplant at 30 days. Differences between the cohorts were expressed as means ± standard deviations and compared with the Student’s t test. Categorial variables were compared with the Fisher exact test.

## Results

### Cardiac energetics and the physiological basis of advanced hemodynamic parameters

The hemometabolic state of a patient defines energy expenditure relative to energy stores. When the transfer of energy from the ventricle to the blood elements during ejection is efficient, energy expenditure is minimized, and finite energy reserves are preserved. We can define the efficiency of this energy transfer as the mechanical efficiency of the heart (19, 20). Mechanical efficiency represents the proportion of the heart's total energy expenditure that is directly responsible for ejection of blood during systole. The work of ejection is defined as the SW and it can be represented on the pressure-volume (PV) curve as the area contained in the PV loop itself. The total energy expenditure of the heart can be represented by the area contained by the boundary of the end-systolic pressure–volume relationship (ESPVR) and end-diastolic pressure–volume relationship (EDPVR) and is referred to as the pressure–volume area (PVA). PVA is equal to the sum of SW and PE. As the heart becomes more loaded and uncoupled, the proportion of total energy expenditure (PVA) that is directly applied to systolic ejection (SW) decreases and more energy is needed to prepare the heart for ejection (PE), which includes the energy needed for calcium handling and myofilament positioning, leading to a reduction in mechanical efficiency (20). Mechanical efficiency is the ratio of SW to total energy expenditure and thus can be defined as:

$$\mathrm{Mechanical}\;\mathrm{Efficiency}\;=\;\frac{\mathrm{Stroke}\;\mathrm{Work}}{\mathrm{Stroke}\;\;\mathrm{Work}\;+\;\mathrm{Potential}\;\mathrm{Energy}}$$

From the above relationship, it can be concluded that efficiency will decrease when the rate of change of PE outpaces the rate of change of SW. At loading conditions beyond this point, mechanical efficiency decreases as potential energy increases out of proportion to stroke work. At the cellular level, this represents a point where the enthalpy of cross-bridging of the actin–myosin unit exceeds useful mechanical work (21). From a physiological perspective, mechanical efficiency can be thought of as the ratio of output energy from the heart divided by input energy to the heart. Efficiency is maximal when output far outpaces input and approaches zero as input far outpaces output. From the above equation, it can be determined that the heart is 50% efficient when the input of the heart is twice the output of the heart.

#### Aortic pulsatility index as a surrogate for the coupling ratio and efficiency

**Figure 1:**
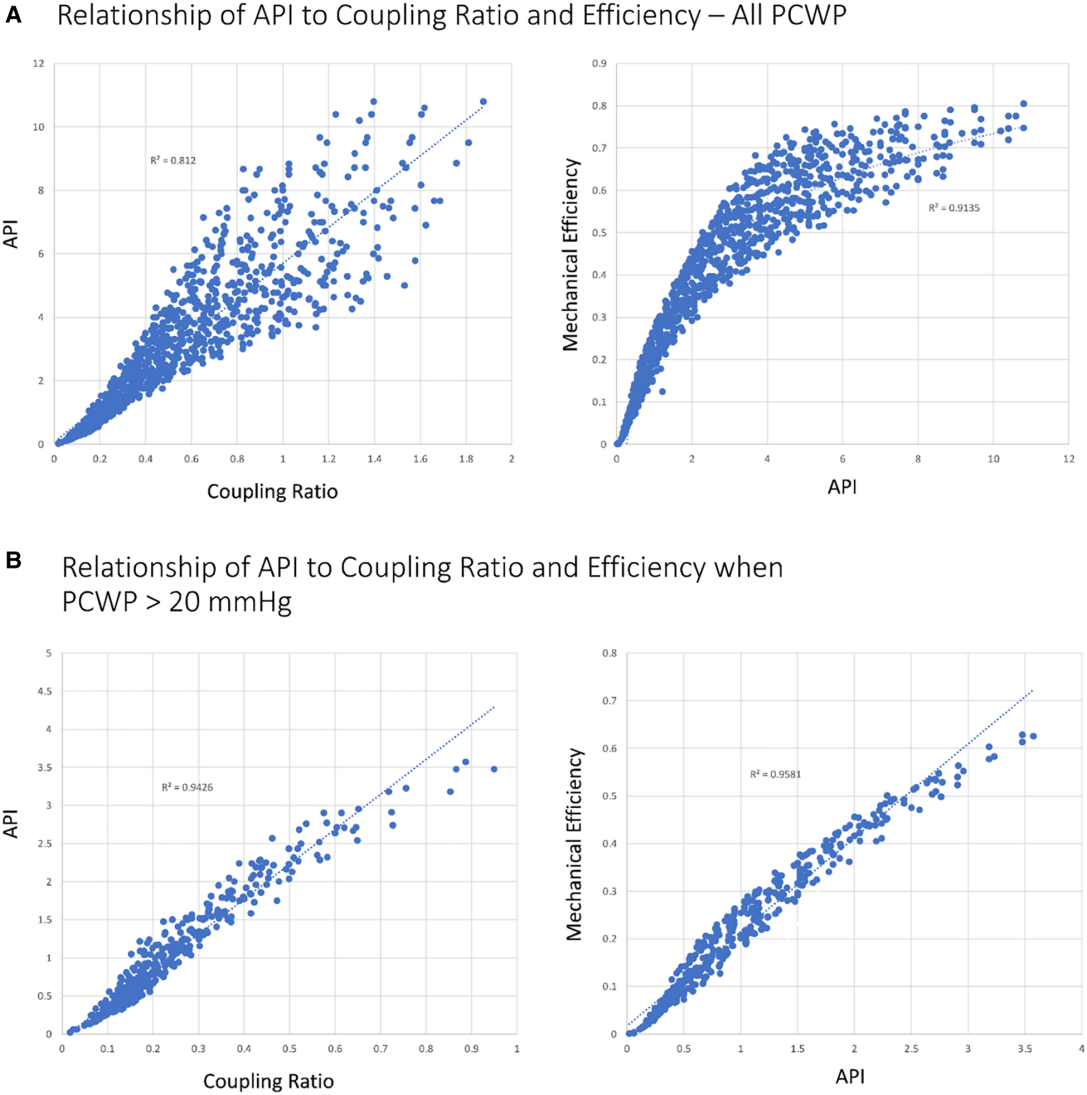
Relationship of API to coupling ratio and efficiency with different loading conditions. (**A**) All simulated scenarios (*N* = 1,010). (**B**) PCWP >20 mmHg (*N* = 399). Coupling ratio, EEs/Ea.

API defined as the aortic pulse pressure divided by PCWP reflects the pulsatile load of ejection on the vasculature for a given ventricular preload and thus mechanistically reflects the performance of the ventriculo-arterial unit. Given that pulse pressure is proportional to ventricular stroke volume, a higher API implies enhanced ventricular output for a given preload and hence is proportional to mechanical efficiency (13). From the above relationship of mechanical efficiency, SW, and PE, API is therefore inversely proportional to PE. The higher the API, the more efficient the transfer of blood and energy from ventricle to vasculature (Figure 1A). The relationships of API to the coupling ratio (*R*^2^ = 0.94) and mechanical efficiency (*R*^2^ = 0.96) become more linear as loading conditions increase (22) (Figure 1B).

#### Cardiac power output and power efficiency

Cardiac power output represents the energy output per unit time of the cardiovascular system. From pressure–volume loops, CPO can be calculated by multiplying ventricular SW by heart rate. CPO can also be estimated as MAP × cardiac output (CO)/451. SW and CPO represent the mechanical work and power of the heart and are directly proportional to total ventricular energy expenditure and myocardial oxygen consumption (MVO2) (20). CPO is a highly prognostic variable in patients in cardiogenic shock after an acute myocardial infarction (AMI) (12). A value of less than 0.6 W (which roughly translates to a MAP of 65 mmHg and CI of 2.2 L/min/m^2^ for the average-sized individual) is often targeted as the minimal acceptable power output when managing patients with shock. While CPO remains highly prognostic in AMI cardiogenic shock (CS-AMI), its prognostic potential in cardiogenic shock secondary to heart failure (CS-HF) has been less consistent (13, 23). If the patient has adequate myocardial reserve, stroke work and thus CPO can be maintained for some time but at the expense of cardiac efficiency and increased intracardiac filling pressures. Therefore, to better conceptualize the prognostic role of CPO, it is necessary to discuss CPO in the context of cardiac efficiency (13, 24).

Given that CPO is the product of SW and HR, we can define the efficiency of power transfer from the ventricle to the vasculature as the power efficiency.

$$\mathrm{Power}\;\mathrm{efficiency}\;=\;\frac{\mathrm{CPO}}{\mathrm{CPO}\;+\;\mathrm{PE}}$$

Similar to mechanical efficiency, when the rate of change of potential energy is greater than the rate of change of external power, power efficiency will decrease.

As discussed previously, API is inversely proportional to PE and this relationship is linear prior to the limits of stretch (Supplementary Figure S1 and Derivations in the Supplementary material). API also happens to be a dimensionless parameter. Similarly, CPO is a derived value with the constant 451 defined to normalize CPO to 1 W for the typical patient with blood pressure 120/80 mmHg, MAP 93.3 mmHg, right atrial (RA) pressure 3 mmHg, and CO 5 L/min (25). Under these same idealized conditions, using a PCWP value of 10 mmHg, API will equal 4 [(120/80 mmHg)/10 mmHg]. Hence, 2/API serves as an estimate of PE prior to the limits of stretch (Derivations in the Supplementary material).

$$\mathrm{Power}\;\mathrm{Efficiency}\;\mathrm{Surrogate}\;=\;\frac{\mathrm{CPO}}{\mathrm{CPO}\;+\;\frac{2}{\mathrm{API}}}$$

By definition, at a Power efficiency of 50%, 2/API = CPO.

$$\mathrm{Power}\;\mathrm{Efficiency}\;(50\text{\%}):\frac{2}{\mathrm{API}}\;=\;\mathrm{CPO}$$

#### Myocardial performance profile and the myocardial performance score

**Figure 2:**
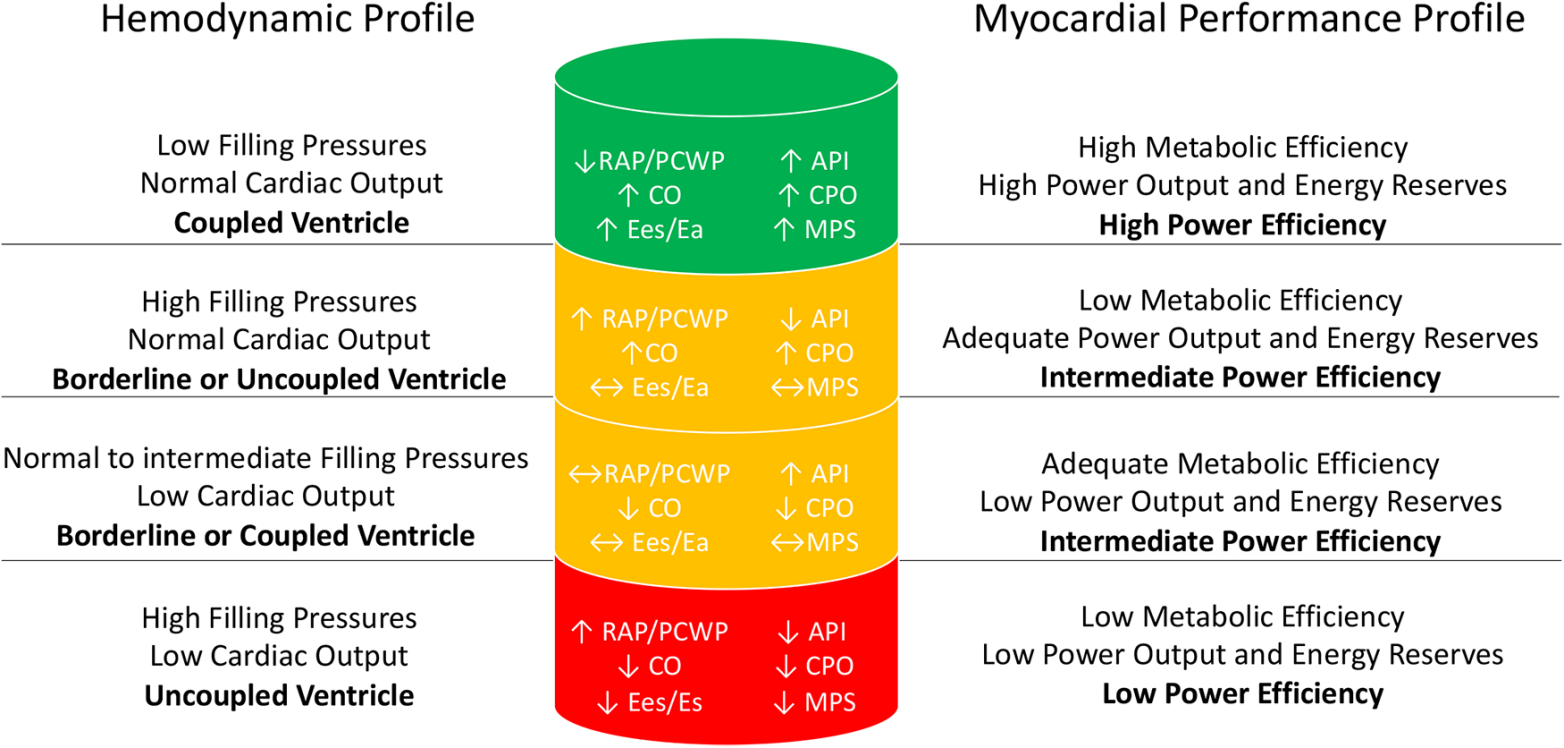
Comparison of the hemodynamic profile and the myocardial performance profile.

Taken together API and CPO provide additive information to help define the hemometabolic state of a given patient that can be described in terms of the myocardial performance profile (Figure 2). CPO represents the energy expenditure of the heart and API represents the efficiency of energy handling. A high-performing heart, similar to a high-performing combustion or electric engine, is able to generate maximum power with high efficiency. Thus, the ideal myocardial performance profile is one that maximizes efficiency and ventricular coupling (high = API) while at the same time is able to maintain an adequate CPO. The power–efficiency relationship or overall myocardial performance can be represented by combining API and CPO into a singular variable called the MPS. Given the inverse relationship of API to PE, the product of CPO and API represents the ratio of useful external work to potential energy.

MPS represents the combined stress of the vasculature and ventricle normalized by PCWP and thus is inversely proportional to the degree of stress for the system for a given strain. The MPS therefore is a marker of ventricular and vascular stretch. As loading conditions increase, ventricular stretch leads to increase in myofilament stretch leading to a drop in efficiency and a rise in potential energy (21). MPS, similar to API, is thus also inversely proportional to PE.

We can arbitrarily define an MPS of 1 as the point where power output and potential energy are equal, or in other words, when power efficiency is 50%. As discussed previously, the calibrated API where PE = CPO for the LV is 2/API.

Thus, the myocardial performance score for the LV is

$$\mathrm{MPS}(\mathrm{LV})\;=\;\frac{\mathrm{CPO}\;\times \;\mathrm{API}}{2}$$

**Figure 3:**
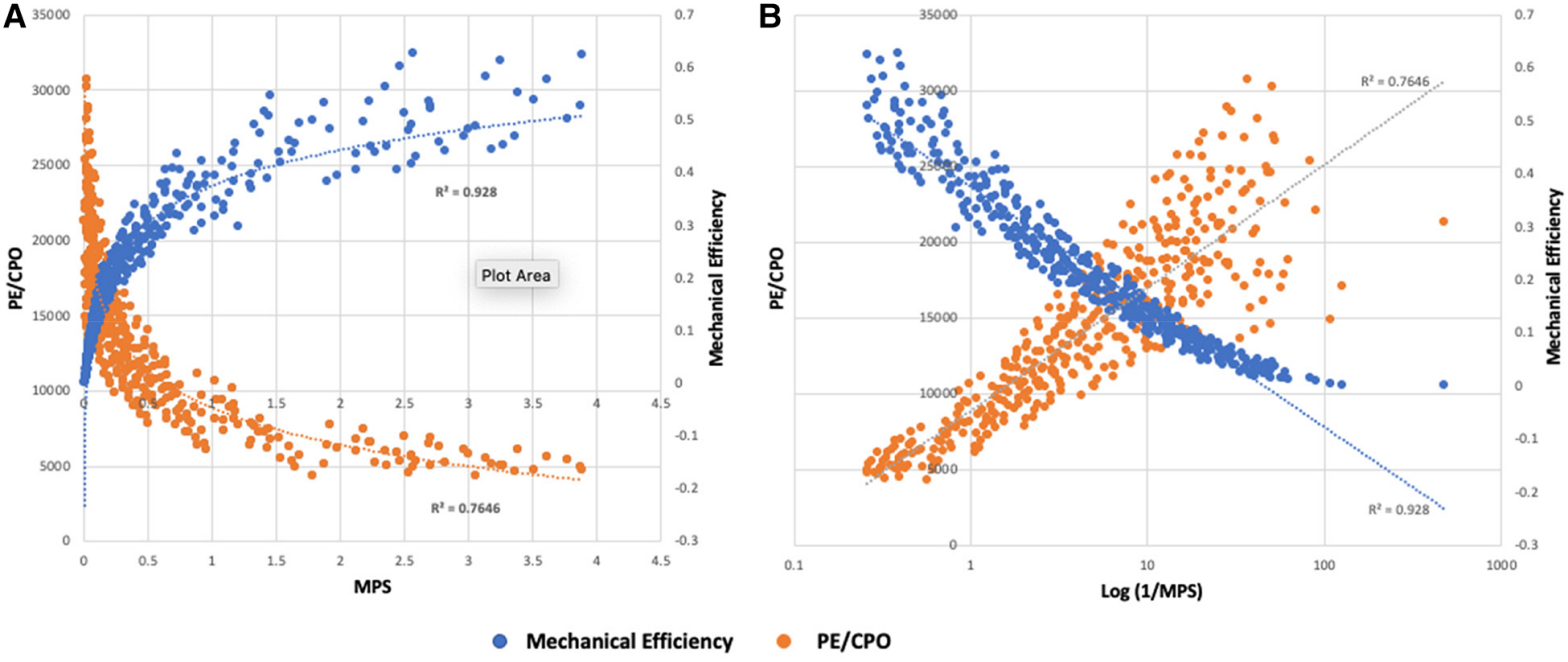
(**A**) Relationship of MPS to mechanical efficiency (SW/PVA) and potential energy normalized to power output (PE/CPO) for simulated scenarios resulting in PCWP >20 mmHg (*N* = 399). Rate of change of mechanical efficiency and PE is largely linear for MPS >1. There is a deceleration of efficiency when MPS 0.5–1 and a more pronounced drop in efficiency at an MPS <0.5. (**B**) Relationship of the log-transformed 1/MPS with mechanical efficiency and PE/CPO. As myocardial stretch increases, 1/MPS increases leading to decrease in mechanical efficiency and increase in PE.

**Figure 4:**
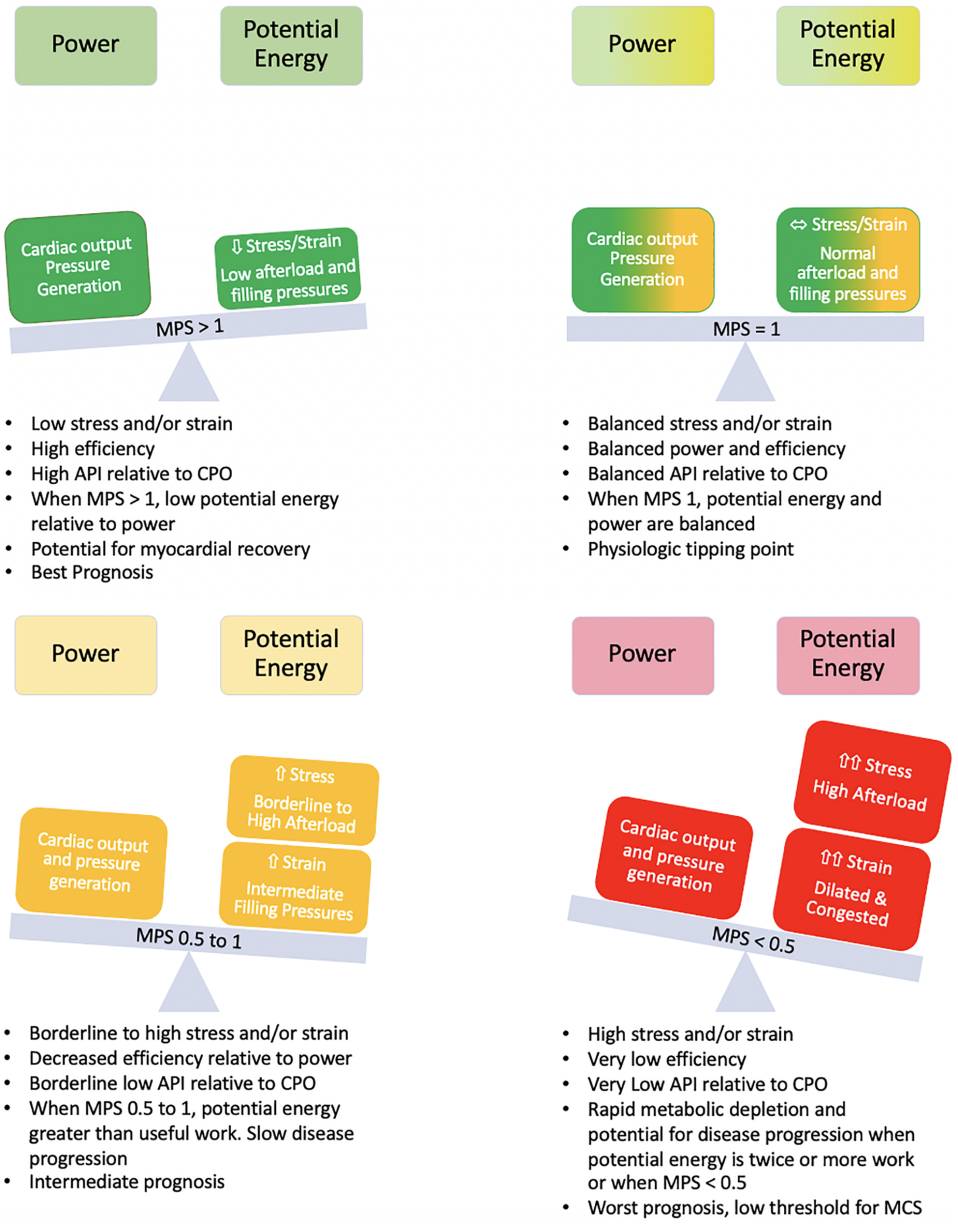
Balance of power and potential energy by MPS. As stress and strain increase, potential energy increases and there is a decrease in efficiency. The rate of change of potential energy relative to power output is linear up until an MPS of 1 but increases relative to power output at an MPS <1 with a further acceleration when MPS <0.5. The high demand on the system at this point can accelerate disease states.

At an MPS of 1, power efficiency is 50% and represents the point of balance between power and efficiency. At an MPS of 2, the efficiency relative to power is maximum and at an MPS <0.5, which corresponds to a power efficiency of 33.3%, there is a rapid decline in efficiency (Figures 3, 4).

#### API, CPO, and MPS in the interpretation of the Starling curve

**Figure 5:**
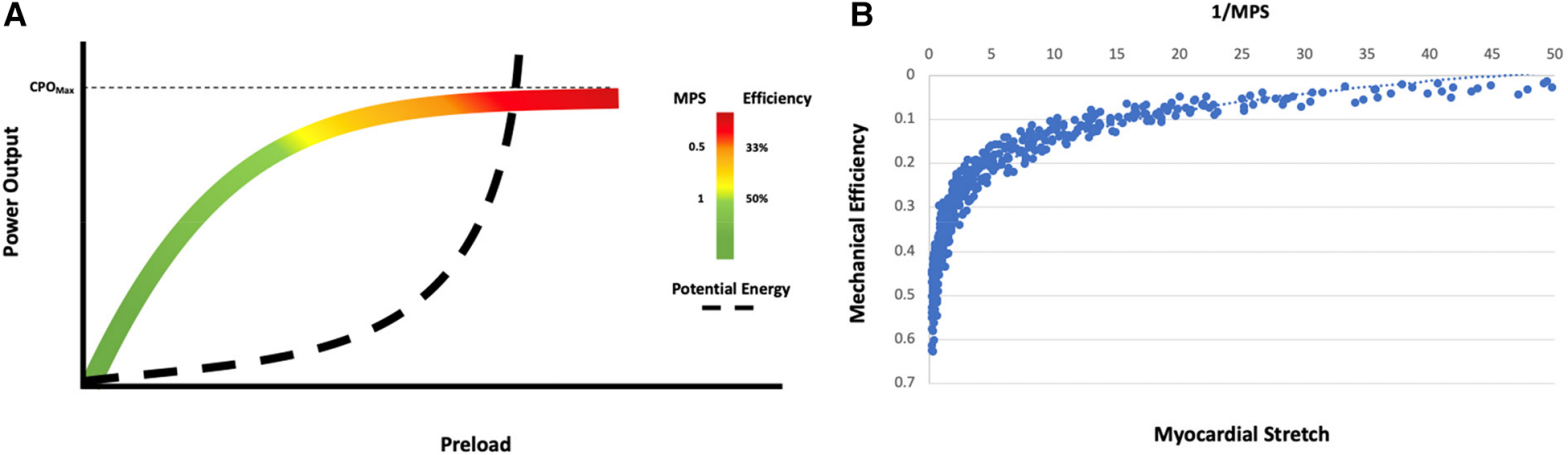
Energetic modeling of the starling curve. (**A**) Interpretation of the starling curve in the context of power output, potential energy, efficiency, and the MPS. The Zone of energy recovery is denoted in green and corresponds to an MPS >1 (>50% power efficiency). Zones of borderline (yellow, MPS 0–1) and rapid (red, MPS <0.5, power efficiency <33.3%) energy depletion are also noted. (**B**) The relationship of mechanical efficiency to 1/MPS in simulated scenarios resulted in PCWP >20 mmHg. Mechanical efficiency decreases as myocardial stretch increases.

If a Starling curve is created with PCWP as the input variable and CPO as the output variable, the ideal metabolic performance score for the patient can be defined (Figure 5). MPS is inversely related to stretch and hence reflects the degree of rightward shift on an individual starling curve (Figures 5A,B). The more rightward shifted one is on a particular starling curve, the lower the MPS and power efficiency and the more uncoupled the ventricle. Power matches potential energy (50% efficient) at an MPS of 1 with an accelerated rise in potential energy and drop in efficiency at an MPS of 0.5. Patients with an MPS of <0.5 are operating on the flat part of their respective starling curves and the ventricle is uncoupled to the downstream circulation. When the MPS is 0.5–1, the patient is still in a modest energy depletion mode with a higher potential energy than power output, and when the MPS is >1, the patient is operating in a high efficiency zone.

### Validation using simulated patients: Harvi modeling of the myocardial performance score

Using Harvi, 1,010 scenarios of patients with varying degrees of heart performance were created by varying Ees, Ea, blood volume, and HR and recording the hemodynamic output. The scenarios were then sorted based on the coupling ratio. Approximately 126 of the 1,010 scenarios established a coupling ratio >1 (fully coupled), 167 scenarios returned a coupling ratio of 0.7 to 1 (borderline coupled), and 718 scenarios returned a coupling ratio <0.7 (uncoupled). In the coupled group, all patients had a PCWP ≤20 mmHg and API >3. A CPO <0.6 W occurred in 12 patients (9.5%) and all were associated with high-output, low-vascular-tone states (high-output heart failure). Median MPS for the coupled group was 4.0 (IQR 3.23–4.66). In the borderline coupled group, 159 of the 167 scenarios (95.2%) had a PCWP ≤20 (all cases ≤23 mmHg) and all patients had an API >2.5. A CPO <0.6 W occurred in 7 of the 167 patients (4.2%). Median MPS for the borderline coupled group was 3.2 (IQR 2.67–3.93). By contrast, among the uncoupled scenarios, 392 of the 718 scenarios (54.6%) had a PCWP >20 mmHg and 307 of the 718 scenarios had a CPO <0.6 W (42.8%). Median MPS was 0.61 (IQR 0.16–1.83) for the uncoupled group. When the uncoupled group was restricted to those with a PCWP >20 mmHg and CPO <0.6 W (228 scenarios), all patients had an API <2 and an MPS <0.5. Under these conditions, API was able to predict the coupling ratio with an *R*^2^ of 0.95 (Figures 1B, 5).

For the entire cohort, an MPS of <0.5 was able to predict a CPO <0.6 W and coupling ratio of <0.7 with Sn of 87%, Sp of 91%, PPV of 81%, and NPV of 94%. Conversely, an MPS >1 was able to predict a CPO ≥0.6 W and coupling ratio ≥0.7 with an Sn of 81%, Sp of 97%, PPV 98%, and NPV 68%. In summary, categorizing a patient according to their MPS (high power efficiency: MPS >1; low power efficiency: MPS <0.5) can help with additional risk stratification and prognostication.

### Clinical validation

As a proof of concept, the performance of the MPS to predict clinically meaningful endpoints was assessed in a cohort of 224 patients with AHA/ACC Stage D heart failure who presented with SCAI Stage C shock. The average age of this cohort was 57 years (48–66 years), 66.5% were men and 39.3% white. All patients in this cohort were deemed to have inadequate perfusion (mean CI 1.8 L/min/m^2^) and unacceptable levels of congestion (mean PCWP 27 mmHg) warranting inotropic infusion with the initiation of milrinone in the cardiac catheterization laboratory. All patients underwent a complete hemodynamic assessment at baseline and then again 10 min after a 50 μg/kg/min milrinone load.

**Figure 6:**
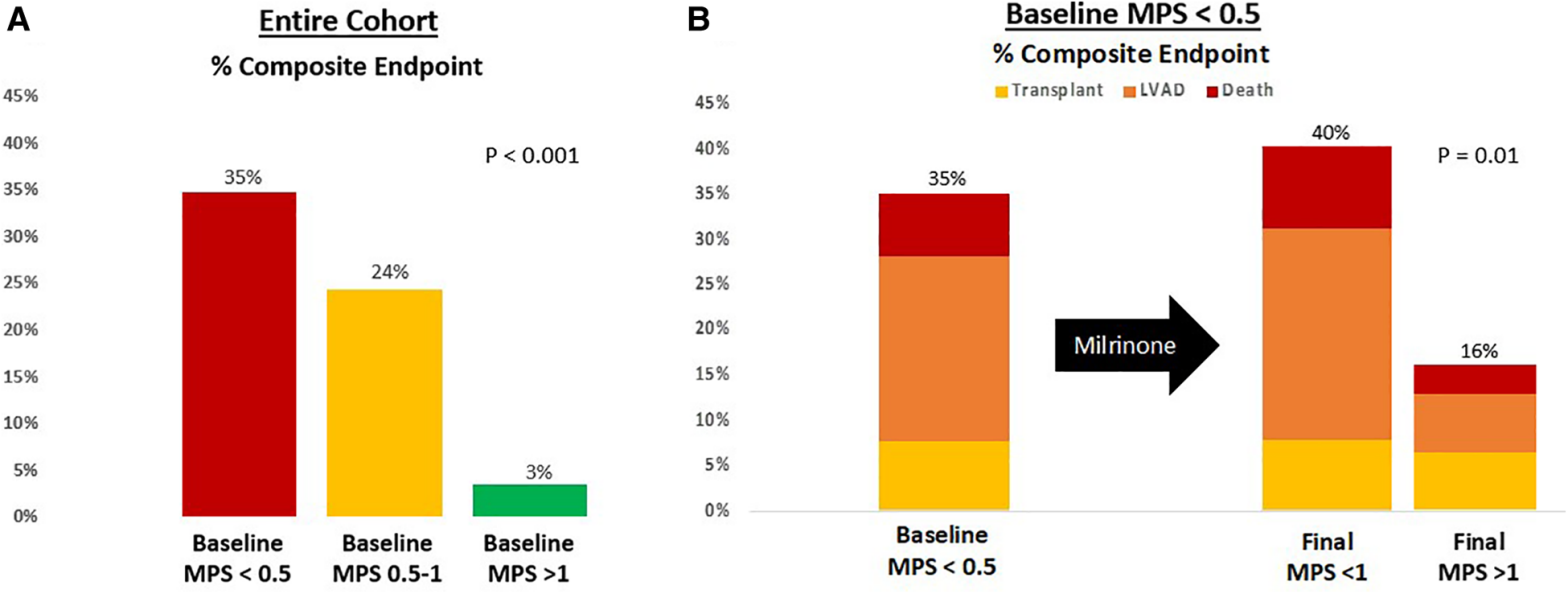
Risk stratification based on (**A**) baseline MPS and (**B**) final MPS after milrinone infusion. The composite endpoint is survival at 30 days free from LVAD or transplant.

The MPS was able to add additional risk stratification. Patients with a baseline MPS of <0.5 had a 35% rate of death, LVAD, or transplant at 30 days, whereas those with an initial MPS >1 had only a 3% rate of the combined endpoint and those with an MPS 0.5–1 had a 24% event rate (*P* < 0.001) (Figure 6A). When the sickest cohort was reexamined after milrinone infusion, those who were able to augment their MPS from an initial score of <0.5 to a final score >1 had a 16% rate of the combined endpoint vs. a 40% event rate for those who had insufficient augmentation (Figure 6B).

## Discussion

The efficient utilization of finite energy reserves is of the utmost importance to promote recovery and/or stabilization. A ventricle that is coupled to the downstream circulation is capable of efficiently utilizing energy stores. The ventriculo-arterial unit is said to be coupled when the contractile ability of the ventricle (Ees) is matched by the ability of the circulation to accept the ejected blood, which is determined by elastance of the vasculature (Ea). In unstressed conditions with normal ventricular function, Ees is greater than Ea with an Ees/Ea (coupling ratio) of 1.5 to 2.0, which maximizes mechanical efficiency. As ventricular function worsens (decrease in Ees), the heart sacrifices efficiency in an attempt to maintain adequate cardiac output and tissue perfusion (26). Activation of the renin-angiotensin-aldosterone (RAAS) system leads to retention of salt and water, increasing effective circulatory and ventricular preload. RAAS activation also causes arteriole vasoconstriction, which increases vascular tone and Ea (19). As a result of this remodeling process, Ees/Ea decreases, the ventricle shifts downward and rightward on the PV curve and tends to operate on the flatter portion of the starling curve. By doing so, the heart is able to maximize the output but it comes at the expense of efficient energy handling (21).

Here it is shown that the physiological inflection point when the efficiency of energy transfer precipitously falls can be predicted using the simultaneous assessment of API and CPO, which can be better understood by incorporating these two complex variables into a single entity called the MPS. As input power increases, over-stretching of the actin–myosin filaments occurs leading to inefficient cross-bridge formation and excess cross-bridge heat (21). Concurrently, the collagen and elastin components of the vasculature are also stretched leading to a decrease in vascular compliance (27). When these changes occur, the rate of change of potential energy (which in turn reflects excess heat production at a cellular level from inefficient cross-bridging and from wavelet reflections in the aorta impeding antegrade flow), outpaces power output and the efficiency of the system drops rapidly. This occurs when the MPS is <0.5. This is the point of maximal rate of change of efficiency and represents the terminal stages of heart failure. The system is perfectly balanced in terms of power output and potential energy when the MPS is 1 and patients enter an energy recovery phase when the MPS >1.

The importance of patient-specific thresholds for decompensation or recovery has been known for some time. Using a conductance catheter that can simultaneously measure ventricular volume and pressure, patient-specific pressure–volume loops can be derived and important information on myocardial energetics and the coupling ratio can be ascertained. While measuring PV loops and recording myocardial energetics and Ees/Ea have immense clinical potential and can provide vital information on the physiological tipping point of a patient, in practice adoption of the PV loop into clinical practice has been slow and it is mostly used as a research tool. Limitations to more widespread use include the technical nature of data acquisition, the high cost of the conductance catheters and other specialized equipment, and the static nature of the assessment. PV loops are not designed for long-term monitoring and thus when used, they only provide a snapshot in time of the patient's cardiac milieu. Less invasive and less costly techniques such as the single-beat approach can be used, but these approaches require computer extrapolations of maximum ventricular pressure and similarly have not been widely adopted (24). Conversely, the myocardial performance score is a user-friendly, cost effective parameter derived from a standard Swan-Ganz catheter, which serves as a powerful prognostic parameter and also provides vital information on myocardial energetics.

The current heart transplantation allocation system has been criticized by the lack of objectivity leading to a large number of exceptions (28). A central driver of the large exception rate is that the current hemodynamic thresholds (SBP < 90 mmHg, CI <1.8 or conditionally <2.0 L/min and PCWP > 15 mmHg) used in the heart allocation system are largely subjective and lack clinical data to support their correlation with waitlist mortality in several patient populations (28, 29). By simultaneously representing power and efficiency, the MPS is a single continuous variable that can add objectivity to the risk assessment. Furthermore, given that the MPS represents myocardial energetics, with a score of <0.5 representing accelerated energy consumption, this hemodynamic variable can be used to assess ongoing stability on the current level of support and can help guide escalation and de-escalation of support when appropriate.

## Limitations

The current analysis is a physiological derivation of the limits of cardiac performance and was validated by the simulated heart failure scenarios using the Harvi application. A proof-of-concept clinical validation was provided; however, a large volume, prospective clinical validation is still needed. As discussed previously, the association of API and MPS with efficiency, the coupling ratio, and power efficiency, respectively, increases with elevated PCWP when the influence of Ea on pulse pressure becomes more impactful. The majority of the patients with advanced heart failure, however, have elevated filling pressures. Finally, here we show that 2/API can be used to represent PE and we assume that the relationship is linear. In accordance with Hooke's law, such an assumption is fair at low to moderate filling pressures when the rate of change of power output follows first-order kinetics and is linear to loading conditions, but 2/API would underestimate PE at higher filling pressures.

## Conclusions

The novel hemodynamic variable, the myocardial performance score, represents the patient-specific power-to-efficiency ratio. At the limits of ventricular and vascular stretch, which occurs at an MPS of <0.5, potential energy rises, and efficiency drops precipitously leading to an abatement of further power output from the heart. The MPS score represents an objective assessment of the energetic state of patients with advanced heart failure.

## Data Availability

The raw data supporting the conclusions of this article will be made available by the authors, without undue reservation.
