# Supplementary material for: Advanced hemodynamics for prognostication in heart failure: the pursuit of the patient-specific tipping point
Source: Front Cardiovasc Med. 2024 Mar 4;11:1365696. doi: 10.3389/fcvm.2024.1365696 (PMC10944906; doi:10.3389/fcvm.2024.1365696)
Supplement: Supplementary file 1 [file Datasheet1.docx]

Mechanical efficiency is the ratio of energy output to energy input. At the cellular level the energy input of the myofilaments is the sum of the activation heat (Q_A_), crossbridge heat (Q_XB_) and external work (W). Q_A_ is a constant and independent of loading conditions whereas W and Q_XB_ will increase with increasing preload or afterload (21). Thus, efficiency can be represented as:

$$Eff=\frac{W}{QA+W+QXB}$$

As preload increases, Q_A_ becomes small relative to W and Q_XB_ and the relationship can be simplified as:

$$Eff=\frac{W}{W+QEB}$$

When the rate of change of crossbridge heat is greater than the rate of change of work, efficiency will decrease. Said another way, when the rate of change of energy input exceeds the rate of change of energy output, efficiency will drop.

At the macro level, energy input is the sum of stroke work and potential energy whereas energy output is equal to stroke work. Since CPO is SW x HR, we can define the power efficiency (P_Eff_) as:

$$Peff=\frac{CPO}{CPO+PE}$$

When the rate of change of potential energy is greater than the rate of change of external power, power efficiency will decrease.

As discussed in the main manuscript, PE is inversely proportional to API times a constant (K_LV_). In accordance with Hooke’s law, this relationship follows first order kinetics before the limits of stretch and thus is linear.

We can define P_Eff_ for the left ventricle as follows:

$$Peff(LV)=\frac{CPO}{CPO+\frac{KLV}{API}}$$

API is a dimensionless parameter. To calibrate the system, we need to define K_LV_ for the left ventricle. If we define the balance point for the system as the point where useful work equals the potential energy, P_Eff_ at this point would be 50%. CPO is a derived value with the constant 451 defined to normalize CPO to 1 watt for the typical patient with blood pressure 120/80 mmHg, MAP 93.3 mmHg, RA pressure 3 mmHg and CO 5L/min (25). Under these same idealized conditions, using a PCWP value of 10 mmHg, API will equal 4 [(120/80 mmHg)/10 mmHg]. The healthy human heart has a Ees/Ea of ~ 2 which said another way has a mechanical efficiency of 66.67% or an energy output which is twice potential energy(34, 35).

We can calibrate the left ventricular power efficiency equation using the normalized values of CPO and API.

We can then define K_LV_ as follows:

$Peff(LV)=\frac{CPO}{CPO+ PE}$ = 66.67% or 2/3

$Peff(LV)=\frac{CPO}{CPO+\frac{KLV}{API}}$ = 66.67% or 2/3

$Peff(LV)=\frac{1}{1+\frac{KLV}{4}}$ = 2/3

Solving for K_LV_:

$$KLV=2$$

We now can estimate P_Eff_ for the left ventricle as follows:

$$Peff\left( LV \right) Surrogate=\frac{CPO}{CPO+\frac{2}{API}}$$

And at a Peff of 50%, 2/API = CPO.

The power efficiency surrogate linearly correlates with both mechanical efficiency and potential energy when power efficiency is greater than 33.3% (which correlates with an MPS of 0.5). At power efficiencies of less than 33.3%, 2/API underestimates true PE and the relationship of the power efficiency surrogate to mechanical efficiency and PE/CPO becomes curvilinear (Supplemental figure 1).

Supplemental Figure 1. Relationship of the power efficiency surrogate with mechanical efficiency and PE/CPO.
